# Address

**Published:** 1871-01

**Authors:** F. H. Rehwinkle

**Affiliations:** President


### ADDRESS

BY F. H. REHWINKLE, PRESIDENT.

Delivered before the Ohio State Dental Society.

*Gentlemen of the Ohio State Dental Society:*—In offering the following remarks at the present time, instead of at the close of my official term, I deviate from the usual custom—but follow in the footsteps of my immediate predecessor, upon reflection, concurring with him in the opinion that any suggestions or statements concerning the general interest and welfare of our profession, are perhaps better timed at the opening, than at the close of our session.

In reviewing the past year, it is gratifying to feel and know that the condition of Dentistry in this State, is in the main highly satisfactory as regards the general spirit of onward and upward endeavor. A commendable emulation manifests itself, even in quarters least expected. A general effort and determination to advance and improve is visible, and encourages the hope that with judicious action on the part of this body, the good work of regenerating the profession from the somewhat demoralized state of former years, may finally be accomplished.

In addition to the regular order of business, of which an excellent programme has been presented to you by your executive committee, I would invite your attention to some other items which, in my opinion merit your serious consideration.

In the first place, would it not be eminently proper to unite your efforts with those of other societies, memorializing Congress for the appointment of competent Dental Surgeons to both Army and Navy?

The want of general information among the people at large, as regards the anatomy and physiology of the teeth, their development, time, and order of irruption, etc., induces me to respectfully suggest to you the duty of laying this matter before the State Board of Education, inducing them by facts and arguments to add to the text books of our common schools, a short and comprehensive chapter on this subject, together with the duty and importance of preserving the teeth, and the best means of attaining that end. The benefit of this to the rising generation would be almost incalculable. Let me, therefore hope the subject will meet with a due share of attention.

The gross carelessness (and in some cases ignorance) of some of the medical practitioners in regard to the influences which certain conditions of the system have upon the fluids of the mouth, and subsequently their effects upon the teeth; also the utter indifference as to the best means of counteracting injurious influences, would seem to make this a proper subject for your consideration. Would it not be well to present a memorial to the medical profession, calling attention to the following subjects and entreating its hearty co-operation:

1. That more general attention be paid to the mouth and teeth of their female patients during the period of gestation, and, by the timely correction of acidulated and viscid fluids of the buccal cavity, preventing in a great measure the rapid decay to which the teeth during this period are especially liable.

2. That advice be given to the nursing mother in regard to her diet, so that a proper and sufficient amount of bone-making material may be secured to the offspring.

*Last.* That in all cases where acids, or *tonics combined with acids* are prescribed, though they may be taken through quills or glass tubes, shall be preceded and followed by alkalies, used as mouth washes.

You may feel that this is a delicate subject, and our cousins of the medical profession may possibly think it presumptous to offer any suggestions, nevertheless, we must not by this, be deterred from doing what might be the means of accomplishing much good. Those of the medical gentlemen to whom these suggestions are entirely superfluous, know too well that there are many, very many practitioners in their ranks, to whom the foregoing hints and suggestions are altogether apropos.

Thus far, the compliance with the provisions of the law regulating the practice of Dentistry in the State of Ohio, has not equaled the expectations of its upholders; and whilst it is to be regretted that the contemplated proviso was stricken out by the Committees of both Houses of the Legislature, an attempt to change the law itself would be precarious and ill-timed; but the condition of things as they now are, seems to call for some action on your part, to disarm all opposition to this law, which possibly might be made by those whose time of grace is fast expiring. Would it not be an advantage in this connection if some plan were devised by which the Board of Examiners should be enabled to hold its sessions in different localities of the State, offering all possible facilities for those who have heretofore found the distance to Columbus or Cincinnati too great? I have not yet heard of any prosecutions for violations of this law. As it now stands, it is the implied duty of every member to prosecute; but, "what is everybody's business is nobody's business," therefore it would seem to suggest itself as desirable, that you should create a special committee, with an advisory member from each Congressional District, to gather the names of parties who will be affected by said law, or who have already violated it, together with the facts in the cases, and to proceed to enforce the law. Should this not seem advisable, to adopt other measures which, in your opinion will accomplish the same end.

The relations of the profession to the "Goodyear Dental Vulcanite Company" are in all respects unaltered, and with no prospect of a change until May, 1872, when the extension of the Goodyear patent expires and when the Cummings patent will have to stand upon its own merits. I feel it my duty to call to your mind the united efforts made to defeat the exorbitant demands of the Boston monopoly. Many of our brethren were discouraged and seemed to think that the "Dental Vul. Co., of Boston," had been but too successful in the struggle; please bear in mind that the profession of the West and North-west, did not in reality, contest the validity of the "Goodyear patent," but that the Cummings patent was the real point at issue. Had it not been for the Weatherby case in Boston, the question as to the validity of the Goodyear patent, would in all probability never have been contested, and that, as to the validity of the Cummings patent might and would probably have remained in abeyance until the expiration of the Goodyear patent; but inasmuch as the District Court of Massachusetts, with one of the Supreme Judges on the bench, allowed a hearing and rendered a judgment on the two patents combined, it became imperatively necessary to contest the Cummings patent at once, and as long as it was wrapped up in the Goodyear parchment, this had to be unfolded to get at the Bacon rind. You are aware that since that time, no U. S. Judge has allowed a heraing upon the merits of the Cummings patent; Judge Nelson, of New York, declared "he knew nothing of the Cummings patent," and declined to hear arguments upon it in connection with the Goodyear patent. All judicial decisions and judgments, except in the case already cited, are solely on the validity of the Goodyear patent.

Whatever our individual opinion may be as to the merits or demerits of vulcanized rubber as a base for artificial teeth, the State Society as a body, owes to its members to make common cause in the coming fight. There is not the slightest doubt in the minds of those who are well informed upon the subject, of the invalidity of the Cummings patent. Take, therefore, steps in time—again effect an organization, retain counsel, and be prepared for the contest.

Finally, gentlemen, let me entreat you to extend to each other every courtesy in debate; speak to the point on subjects under discussion, bearing in mind that time is valuable and that a great deal of business requires your consideration. Thanking you, gentlemen, for your kind and patient attention, please accept the honest wish of my heart that the most perfect harmony may prevail among us, and that the prosperity and usefulness of this Society may increase day by day and year by year.

